# *In vitro* RNA Cleavage Assays to Characterize IRE1-dependent RNA Decay

**DOI:** 10.21769/BioProtoc.3307

**Published:** 2019-07-20

**Authors:** G. Elif Karagöz, Jirka Peschek, Peter Walter, Diego Acosta-Alvear

**Affiliations:** 1 Max Perutz Labs Vienna, Medical University of Vienna, Vienna, Austria; 2 Department of Biochemistry and Biophysics, University of California, San Francisco, San Francisco, CA, USA; 3 Howard Hughes Medical Institute, USA; 4 Department of Molecular, Cellular and Developmental Biology, University of California, Santa Barbara, Santa Barbara, CA, USA

**Keywords:** IRE1, Unfolded protein response, RNA cleavage, XBP1, Regulated IRE1-dependent decay, ER stress

## Abstract

The kinase/RNase IRE1 is a key effector of the cellular response to endoplasmic reticulum stress. The RNase activity of IRE1 can be measured in cells or in the test tube. Here we describe a protocol for the *in vitro* cleavage and analysis of RNA substrates of IRE1. The method consists of the *in vitro* transcription, purification and re-folding of IRE1 substrate RNAs followed by their cleavage using recombinant cytosolic kinase/RNase domains of IRE1 and the separation of the resulting fragments by denaturing polyacrylamide gel electrophoresis. This protocol allows the study of the cleavage kinetics of IRE1’s RNA substrates *in vitro*.

## Background

**Figure 1.**
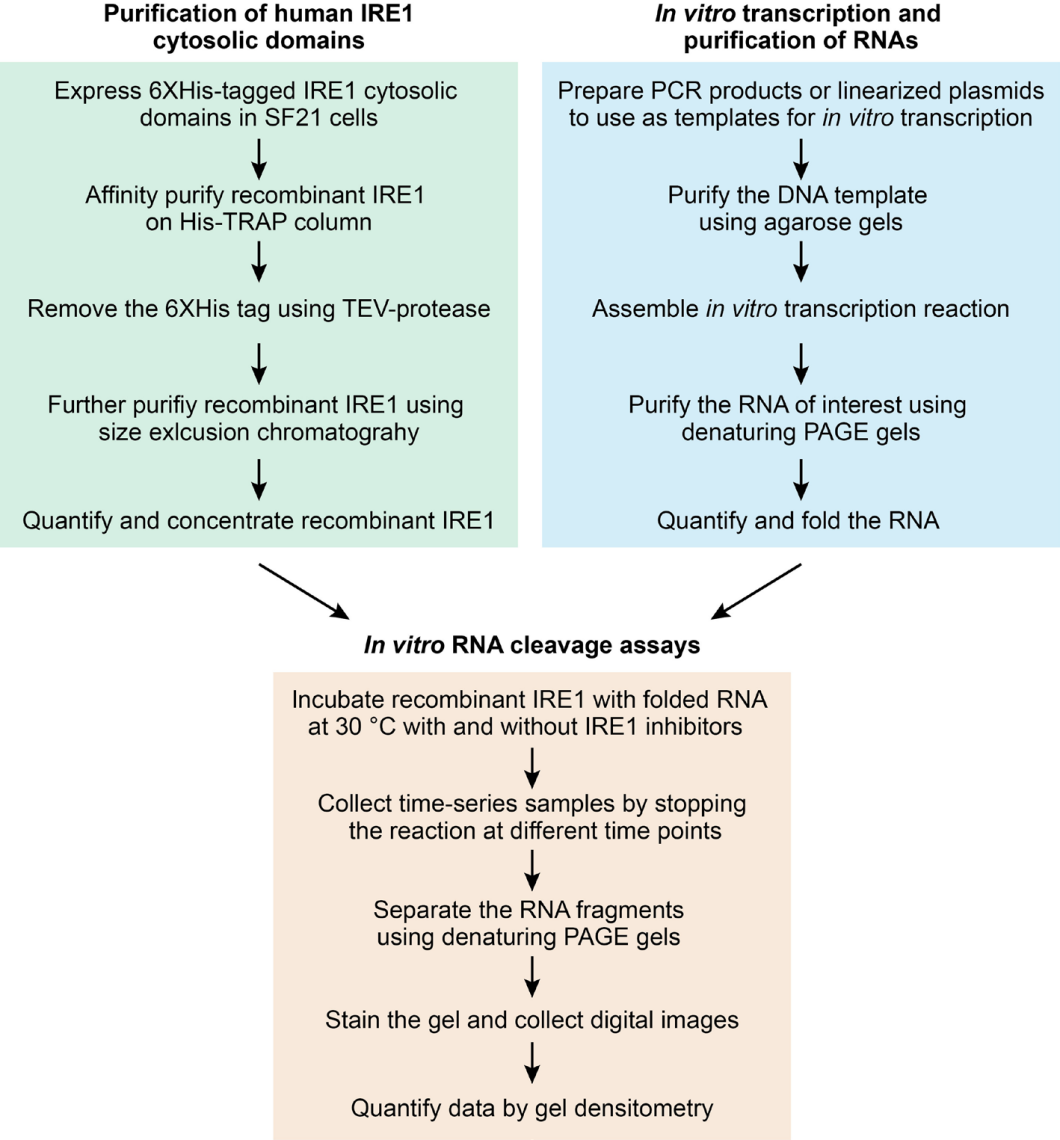
Protocol workflow

Accumulation of un(mis)folded proteins in the endoplasmic reticulum (ER) causes ER stress and activates the unfolded protein response (UPR), an adaptive mechanism that maintains ER homeostasis (Reviewed in Karagöz *et al.*, 2019). IRE1, a transmembrane ER stress sensor/transducer with cytosolic kinase and RNase activities, governs the most conserved UPR signaling arm, and it is found from yeasts to metazoans (Mori, 2009). ER stress leads to IRE1 oligomerization and activation of its C-terminal cytosolic RNase domain. IRE1 preserves ER homeostasis in two ways. First, by its most studied mechanism, IRE1’s RNase domain cleaves an intron found in the *XBP1* mRNA to initiate an unconventional splicing event that activates the transcription factor XBP1 (Yoshida *et al.*, 2001; Calfon *et al.*, 2002; Peschek *et al.*, 2015). Active XBP1^S ^(“S” for spliced) controls the upregulation of genes that improve protein processing capacity of the ER (Lee *et al.*, 2003; Acosta-Alvear *et al.*, 2007). Second, IRE1 degrades ER-bound mRNAs in a process known as regulated IRE1-dependent decay (RIDD), to reduce the ER protein folding load or to initiate specific cytoprotective responses (Hollien and Weissman, 2006; Hollien *et al.*, 2009; Bae *et al.*, 2019). IRE1’s RNase output has been measured on a global scale through microarray-based gene expression analysis in wild-type and in IRE1-deficient cells (Hollien and Weissman, 2006; Han *et al.*, 2009; Hollien *et al.*, 2009; So *et al.*, 2012). While this type of approach lends itself to the discovery of potential IRE1 substrates, it does not allow evaluating the direct engagement of IRE1 with potential substrates. Moreover, such global approaches can be costly and technically demanding. We and others have measured IRE1’s RNase output in mammalian cells using *XBP1* mRNA splicing and RIDD reporter constructs (Iwawaki *et al.*, 2004; Iwawaki and Akai, 2006; Sidrauski *et al.*, 2013; Mendez *et al.*, 2015; Moore and Hollien, 2015; Peschek *et al.*, 2015), or by directly assessing the mRNA levels of endogenous spliced *XBP1* mRNA or of validated RIDD targets by TaqMan and/or RT-PCR assays (Acosta-Alvear *et al.*, 2007; Hollien *et al.*, 2009; Sidrauski *et al.*, 2013; Mendez *et al.*, 2015; Peschek *et al.*, 2015). While useful, these techniques do not allow precise time-resolved measurements of RNA processing by IRE1. Precise kinetic measurements of IRE1’s RNase activity require examining RNA cleavage in the test tube. We and others have measured the kinetics of *XBP1* mRNA cleavage by IRE1 using short RNA substrates containing *XBP1* mRNA stem-loops recognized by IRE1 (Korennykh *et al.*, 2009; Lu *et al.*, 2014; Mendez *et al.*, 2015; Peschek *et al.*, 2015; Acosta-Alvear *et al.*, 2018). In cells, however, IRE1 is presented with cognate RNA structures embedded in full-length mRNAs. Since RNA folding is influenced by the size of the molecule and local sequence composition (Cheng *et al.*, 2015), non-natural short RNA substrates containing putative IRE1 cleavage sites may contribute to false positive or false negative results in *in vitro* RNA cleavage assays. Radioactive *in vitro* cleavage assays for IRE1 RNA substrates of ~400-500 nucleotides in length have been performed (Lu *et al.*, 2014; Tam *et al.*, 2014), and while undoubtedly useful, the use of radioactivity could be limiting for many laboratories. With these considerations in mind, we have developed a non-radioactive protocol for analyzing the cleavage of long RNA substrates of IRE1 *in vitro* (Figure 1). Our method can be performed in the presence and absence of purified ribosomes to account for IRE1 activity in IRE1-ribosome complexes (Acosta-Alvear *et al.*, 2018), it can be deployed to study the RNase activity of IRE1 and its consequences in the context of maintaining cell viability (Lu *et al.*, 2014), and it can be useful for assessing the specificity of recently developed IRE1 inhibitors (Cross *et al.*, 2012; Harnoss *et al.*, 2018).

## Materials and Reagents

- Purification of recombinant cytosolic domains of human IRE1
  1. Superdex 200 10/300 GL size exclusion chromatography column (GE Healthcare, catalog number: 17517501)
  2. 50 ml screw-cap conical tubes (various suppliers)
  3. 5 ml HisTrap HP histidine-tagged protein purification column (GE Healthcare, catalog number: 17524801)
  4. Size exclusion chromatography columns HiLoad^®^ 16/600 Superdex^®^ 200 (GE Healthcare, catalog number: 28989335), or similar
  5. Amicon Ultra-15 centrifugal filters (30 kDa MW cut-off; Mfr. Millipore, catalog number: 903008)
  6. SF21 cells (SFM adapted, Invitrogen, catalog number: 11497-013)
  7. Sf-900^TM^ II SFM (Gibco, Thermo Fisher Scientific, catalog number: 10902096)
  8. Fetal bovine serum (FBS), heat inactivated (Gibco, Thermo Fisher Scientific, catalog number: 10082147)
  9. Antibiotic-antimycotic solution (100x), stabilized (Sigma-Aldrich, catalog number: A5955)
  10. Baculovirus expression construct encoding N-terminal hexahistidine tagged IRE1 of human origin bearing a 43 amino acid long portion of a linker domain located between the epitope tag and the kinase domain (IRE1-KR43, amino acids 521-977) as described in Li *et al.*, 2010
  11. cOmplete EDTA-free protease inhibitor cocktail tablets (Mfr. Roche. Sigma-Aldrich, catalog number: 11873580001)
  12. TCEP-HCl (Sigma-Aldrich, catalog number: 75259)
  13. Tobacco-etch virus (TEV) protease (recombinantly expressed and purified from *E. coli*)
  14. HEPES (Sigma-Aldrich, catalog number: H3375)
  15. KCl, 1 M solution (Sigma-Aldrich, catalog number: 60142)
  16. MgCl_2_, 2 M solution (Sigma-Aldrich, catalog number: 68475)
  17. Imidazole (Sigma-Aldrich, catalog number: I5513)
  18. SF21 cell lysis buffer (see Recipes)
  19. IRE1 dialysis buffer (see Recipes)
- *In vitro* transcription, purification and refolding of IRE1 substrate RNAs
  1. Plastic cling-wrap
  2. Spin-X centrifuge tube filters, pore size 0.45 μm (Mfr. Corning Costar. Sigma-Aldrich, catalog number: CLS8162)
  3. Non-stick 1.5 ml RNase-free tubes (Mfr. Applied Biosystems., catalog number: AM12450)
  4. Non-stick 0.5 ml RNase-free tubes (Mfr. Invitrogen., catalog number: AM12350)
  5. Sterile No. 20 G syringe needles (Mfr. Beckton-Dickinson, catalog number: 305176)
  6. pUC19 (Addgene plasmid #50005 or Thermo Scientific, catalog number: SD0061)
  7. T7 oligonucleotide 5′-TAATACGACTCACTATAG(N_14-21_)-3′; where N corresponds to bases on the target gene of interest to amplify by PCR (Integrated DNA Technology)
  8. Phusion high-fidelity DNA polymerase (New England Biolabs, catalog number: M0530S)
  9. dNTPs solution (New England Biolabs, catalog number: N0447S)
  10. RNaseZAP RNase decontamination solution (Invitrogen, catalog number: AM9780)
  11. QIAquick PCR & gel cleanup kit (QIAGEN, catalog number: 28506)
  12. DNA clean and concentrator-5 kit (Zymo Research, catalog number: D4013)
  13. HiScribe T7 high yield RNA synthesis kit (New England Biolabs, catalog number: E2040S)
  14. RNase-free DNase I (New England Biolabs, catalog number: M0303S)
  15. Novex 6% TBE-Urea PAGE gels (Invitrogen, catalog number: EC6865BOX)
  16. Novex TBE-Urea 2x sample buffer (Invitrogen, catalog number: LC6876)
  17. Novex TBE-Urea 5x running buffer (Invitrogen, catalog number: LC6675)
  18. 3 M NaOAc pH 5.5, RNase-free (Mfr. Invitrogen, catalog number: AM9740)
  19. RiboRuler low range RNA ladder (Thermo Scientific, catalog number: SM1833)
  20. 80% ethanol in RNase-free water
  21. 70% ethanol
  22. Pure isopropanol
  23. RNase-free water (Ambion, catalog number: AM9930)
  24. Recombinant RNasin RNase inhibitor (Promega, catalog number: N251A)
  25. EDTA (Sigma-Aldrich, catalog number: EDS)
  26. HEPES (Sigma-Aldrich, catalog number: H3375)
  27. NaCl (Sigma-Aldrich, catalog number: S3014)
  28. MgCl_2_, 2 M solution (Sigma-Aldrich, catalog number: 68475)
  29. RNA gel extraction buffer (see Recipes)
  30. RNA re-suspension buffer (see Recipes)
- *In vitro* cleavage assay of *in vitro* transcribed RNAs with purified recombinant IRE1 cytosolic domains and denaturing urea-PAGE of IRE1-generated RNA fragments
  1. Non-stick 1.5 ml RNase-free tubes (Mfr. Applied Biosystems, catalog number: AM12450)
  2. Non-stick 0.5 ml RNase-free tubes (Mfr. Invitrogen, catalog number: AM12350)
  3. Purified recombinant IRE1 cytosolic kinase/RNase domains (IRE1-KR43, 50 nM to 5 µM per reaction)
  4. Purified, refolded substrate RNA (25-100 ng per reaction)
  5. TCEP-HCl (Sigma-Aldrich, catalog number: 75259)
  6. Proteinase K solution (Mfr. Invitrogen., catalog number: AM2546)
  7. IRE1 inhibitor III, 4µ8C (CAS 14003-96-4; Calbiochem. Mfr. EMD Millipore. Sigma-Aldrich, catalog number: 212512)
  8. Novex 6% TBE-Urea PAGE gels (Invitrogen, catalog number: EC6865BOX)
  9. Novex TBE-Urea 2x sample buffer (Invitrogen, catalog number: LC6876)
  10. Novex TBE-Urea 5x running buffer (Invitrogen, catalog number: LC6675)
  11. SYBR Gold nucleic acid gel stain (Invitrogen, catalog number: S11494)
  12. RiboRuler low range RNA ladder (Thermo Scientific, catalog number: SM1833)
  13. HEPES (Sigma-Aldrich, catalog number: H3375)
  14. NaCl (Sigma-Aldrich, catalog number: S3014)
  15. MgCl_2_, 2 M solution (Sigma-Aldrich, catalog number: 68475)
  16. Glycerol (Sigma-Aldrich, catalog number: G5516)
  17. Urea (Sigma-Aldrich, catalog number: 51456)
  18. SDS (Sigma-Aldrich, catalog number: L3771)
  19. EDTA (Sigma-Aldrich, catalog number: EDS)
  20. Bromophenol blue (Sigma-Aldrich, catalog number: B8026)
  21. Xylene cyanol FF (Sigma-Aldrich, catalog number: X4126)
  22. 2x RNA cleavage buffer (see Recipes)
  23. Stop solution (see Recipes)

## Equipment

1. Pipettes
-80 °C freezer
3. Sterile No. 11 scalpels (Mfr. Graham-Field, Fisher Scientific, catalog number: 08-927-5B)
4. Cell homogenizer (Avestin Emulsiflex-C3, or similar)
5. Superspeed fixed angle rotor (SS-34, Mfr. Sorvall., Thermo Scientific, catalog number: 28020TS, or similar)
6. Refrigerated benchtop centrifuge (Beckman Coulter Allegra 6R, or similar) equipped with a horizontal swinging bucket rotor (Beckman GH-3.8 or similar), and rotor adaptors for 15 ml and 50 ml conical tubes
7. Benchtop microcentrifuge (Eppendorf 5424, or similar)
8. ÄKTA pure protein purification system (GE Healthcare, catalog number: 29018224), or similar fast-performance liquid chromatography [FPLC] system)
9. Thermomixer C, equipped with a 1.5 ml tube block (Eppendorf, catalog number: 2231000574, or similar)
10. Thermocycler (BioRad C1000, S1000 or similar)
11. Microvolume spectrophotometer (Thermo Scientific, NanoDrop ND-2000; or similar)
12. XCell SureLock mini-cell electrophoresis system (Invitrogen, catalog number: EI0001)
13. Electrophoresis power supply (BioRad PowerPac basic power supply, catalog number: 1645050; or similar)
14. Gel imaging system (BioRad ChemiDoc XRS+, catalog number: 1708265; or similar)
15. UV transilluminator (UVP, catalog number: 95042001; or similar)

## Software

1. ImageJ image processing and analysis software (National Institutes of Health and Laboratory of Optical and Computational Instrumentation, University of Wisconsin at Madison, Madison, WI, USA; https://imagej.nih.gov/ij/)

## Procedure

- Purification of recombinant cytosolic domains of human IRE1
  1. Grow SF21 cells in SF-900 II SFM media supplemented with 10% FBS and 1x antibiotic-antimycotic solution (100 U/ml penicillin, 100 µg/ml streptomycin, 250 ng/ml amphotericin B) at 28 °C in Erlenmeyer flasks, shaking at 150 rpm.
  2. Infect SF21 cells at mid logarithmic growth phase at a density of 1 x 10^6^-2 x 10^6^ cells per ml with high titer baculoviral stock.
  3. Collect the SF21 cells 72-96 h after infection in 50 ml conical tubes and pellet them by centrifugation at 4 °C, at 160 *× g* for 5 min, using a refrigerated benchtop centrifuge (Beckman Coulter Allegra 6R or similar). At this point you can either flash freeze the cell pellet and store it at -80 °C, or continue with the purification of the protein.
  4. Suspend the pellet from 250 ml of SF21 cells (around 5 ml dry volume) in 20 ml ice-cold SF21 cell lysis buffer. Keep the tube on ice.
  5. Lyse the cells by passing them once through the homogenizer. Set the Emulsiflex homogenizer at 16,000 psi. Collect the homogenized cells in ice cooled tubes; keep the homogenates on ice.
  6. Centrifuge the lysate at 30,500 *× g* for 40 min at 4 °C in SS-34 rotor to remove cell debris.
  7. Equilibrate the 5 ml HisTrap HP column with SF21 cell lysis buffer using the FPLC system, which is kept in a cooling cabinet or cold room.
  8. Load the clarified cell lysate onto the 5 ml HisTrap HP column at a flow rate of 2 ml/min to ensure efficient binding of the protein.
  9. Wash the bound protein with 20 column volumes of lysis buffer at a flow rate of 5 ml/min.
  10. Elute the protein with an imidazole gradient (0 to 500 mM) in 15 column volumes in the SF21 cell lysis buffer. Analyze a small sample of each collected fraction by SDS-PAGE to define the fractions in which the protein eluted. Pool the fractions containing the purified protein.
  11. Incubate the purified IRE1-KR43 with TEV protease (250 μl of 0.5 mg/ml) to remove the hexahistidine tag overnight (12-16 h) at 4 °C during dialysis against the IRE1 dialysis buffer.
  12. Load the protein onto a HisTrap HP column equilibrated with the IRE1 dialysis buffer after TEV cleavage to remove impurities as well as the fraction of IRE1-KR43 still containing the hexahistidine tag.
  13. Further purify the protein by running the HisTrap HP column eluate on a HiLoad 16/600 Superdex 200 column (GE Healthcare) which has been equilibrated with the dialysis buffer with 2 column volumes according to the manufacturer’s instructions.
  14. Concentrate the purified IRE-KR43 protein using Amicon Ultra centrifugal filters to 50 μM and flash freeze in 25 μl aliquots. Store at -80 °C.
- *In vitro* transcription, purification and refolding of IRE1 substrate RNAs
  1. Prepare templates for *in vitro* transcription. Assemble multiple parallel reactions to increase yield. The DNA templates can be either PCR products prepared with Phusion polymerase (a high-fidelity DNA polymerase that generates blunt ends), or linearized pUC19 plasmids cut at the 3′ end of the sequence encoding the RNA of interest. We routinely use 1 μg of gel purified DNA templates for *in vitro* transcription. To account for loses during purification, start with at least 2 μg of PCR products or 2 μg of plasmid for digestion.
*
Note: Design oligonucleotides to use as primers to amplify the sequence encoding your RNA of interest from high-quality cDNA obtained from the cell of your choice or from available cDNA clones. Use the T7 oligonucleotide 5′-TAATACGACTCACTATAG(N_14-21_)-3′ as your forward primer and a gene-specific reverse primer. If you want to clone the templates, engineer restriction enzyme recognition sites compatible with the multiple cloning site of pUC19 into your oligonucleotides. Use recognition sites for enzymes that generate blunt-ends or 5′-overhangs on the 3′-end of your cloned DNA of interest. If you are planning on using linearized plasmids as templates for the in vitro transcription reaction, digest the plasmids to completion.
*
  2. Purify the templates from Step B1 above from 1% agarose gels. Use the QIAquick PCR & gel cleanup kit following the manufacturer’s recommendations. Elute DNA from each column with 30 μl of elution buffer.
*Note: Do not skip the gel purification step. Unincorporated oligonucleotides, off-target amplicons and circular plasmids all contribute to the synthesis of heterogenous RNAs.*
  3. Concentrate and further clean-up the gel-purified DNA templates using the Zymo DNA clean and concentrator-5 kit following the manufacturer’s recommendations. Elute DNA from each column with 6 μl of elution buffer. Quantify your template concentration and purity using a microvolume spectrophotometer and adjust the DNA concentration to 500 ng/μl.
*
Note: Highest transcription rates are obtained with highest purity templates. Expect A_260_/A_280_ ratios of ~1.8 and A_260_/A_230_ ratios > 2.0
*
  4. Transcribe RNA using 1 μg of DNA template as input with the HiScribe T7 high yield RNA synthesis kit in 20 μl reactions, for 2 h at 37 °C, following manufacturer’s recommendations.
*Notes:*
    - *While the reactions are running, pre-run a Novex 6% TBE-Urea PAGE gel for 20 min in 1x Novex TBE-Urea running buffer.*
    - *Other pre-cast TBE-urea PAGE gels can be used as an alternative to Novex gels with compatible electrophoresis systems. While we routinely do not cast our own TBE-Urea gels, denaturing TBE-urea PAGE gels prepared in the laboratory could also be used.*
  5. Stop the reactions by adding 2 μl DNase to the reactions, pipette gently to mix and incubate for an additional 15 min at 37 °C.
  6. Add 1 volume of Novex TBE-Urea 2x sample buffer to the reactions, mix well by pipetting and heat at 80 °C for 3 min, then cool the samples immediately on ice. Load your samples on the Novex 6% TBE-Urea PAGE gel from Step B4a “NOTE”. Load 2 μl of diluted RiboRuler low range RNA ladder on the first well. Run the gel using the XCell SureLock mini-cell electrophoresis system at 100 V (constant voltage) for 50-60 min. For longer RNAs run the gel for a longer time (around 90 min) to achieve better separation. The dark blue bromophenol blue dye front corresponds to a fragment length of 25 nt according to the manufacturer’s instructions.
*Notes:*
    - *Before loading your samples, pipette buffer vigorously into each well of the gel to remove urea buildup. Load your samples on the rinsed, clean wells. Use a single gel per transcription reaction; if using a 10-well gel, load ~5 μl of sample per well. Do not overload the gel.*
    - *Dilute the RiboRuler low range RNA ladder in RNase free water to load 10-20 ng RNA per band. The undiluted RNA ladder contains 140 ng of each RNA transcript.*
  7. Disassemble the XCell SureLock mini-cell, remove the gel and dismantle the gel cassette carefully, leaving the gel mounted on one of the plastic plates. Stain the gel with SYBR Gold (diluted as per manufacturer’s recommendations in 1x TBE) by submerging the gel in the buffer for 20 min at room temperature.
*Note: Avoid touching the gel with gloved fingers as it will leave marks. We have found that staining the gel without agitation yields better results. Cover the gels to prevent exposure to light while staining.*
  8. Carefully remove the gel from the backing plate and place it on plastic cling-wrap. Wipe the stage of the UV transilluminator with RNaseZAP RNase decontamination solution and follow by wiping the surface with 70% ethanol. Place the gel on the plastic cling wrap on the transilluminator and visualize the RNA. Use a sterile No. 11 scalpel to cut around the band of interest. Collect the band of interest in a non-stick 0.5 ml RNase-free tube. Use one tube for every 2-3 lanes of gel (max. gel slice per tube should not exceed 1 mm x 15 mm x 5 mm).
*Notes:*
    - *It is possible to have too much RNA loaded on the gel, in which case expect to see “ghosts” around the band of interest (regions where the dye accumulates around the band). Such event does not compromise the RNA extraction downstream.*
    - *Do not drag the tip of the scalpel over the transilluminator stage. Instead place the blade edge parallel to the surface and press down around the band. No. 11 scalpels have straight blade edges.*
  9. Nest each 0.5 ml tube containing the gel fragments inside a non-stick 1.5 ml RNase-free tube. Carefully pierce the bottom of the 0.5 ml tube with a sterile 20 G syringe needle. Do not pierce the larger 1.5 ml tube. Spin in a benchtop microcentrifuge at maximum speed for 3 min to force the gel through the hole. Transfer any residual gel from the 0.5 ml tube to the holder tube. Discard the 0.5 ml tubes.
  10. Add ~3 gel volumes of RNA gel extraction buffer to the crushed gel in the holder tubes. Incubate for one hour at room temperature with constant agitation at 1,000 rpm in a thermomixer.
  11. Remove tubes from thermomixer. Vortex the gel slurry and recover it completely using a cut P1000 tip. Transfer the slurry to a Spin-X centrifuge tube filter. Be careful when transferring as gel pieces can clog the pipette tip. Spin the Spin-X centrifuge tube filters at max speed in a benchtop microcentrifuge for 3 min. The filtrate containing your RNA will be at the bottom of the filter tube and the gel pieces will stay on the filter.
  12. Transfer the filtrate to a fresh non-stick nuclease free 1.5 ml microcentrifuge tube. Pool all filtrates for the same RNA (up to ~0.5 ml aliquots). Add 1/9 of the volume of 3 M NaOAc pH 5.5 and 1 volume of isopropanol and incubate for at least 30 min at -80 °C to precipitate the RNA.
  13. Spin at maximum speed in a benchtop microcentrifuge at 4 °C for 30 min top pellet the RNA. Remove the supernatant being careful not to disturb the pellet. Wash the RNA pellet twice with 1 ml of 80% ice-cold ethanol prepared in RNase-free water.
  14. Remove ethanol from the pellet. Spin the tubes to collect trace ethanol from the walls of the tube and remove as much as possible of the remaining ethanol with a pipette. Air-dry the RNA pellets for at least 10 min at room-temperature. Make sure all ethanol has evaporated before proceeding. Resuspend the pellets in 20 μl RNase-free water or RNA resuspension buffer by gentle pipetting.
  15. Fold the RNAs from Step B14 by incubating them for 5 min at 95 °C in a thermocycler and then slowly cool the samples (1 °C/min) until they reach 25 °C. Keep the samples on ice and use immediately for *in vitro* cleavage assays or store them at -80 °C for future use.
*
Note: Before setting up the in vitro cleavage assays, measure the RNA concentration and purity using a microvolume spectrophotometer. Expect to obtain A_260_/A_280_ ratios ~2.0 and A_260_/A_230_ ratios > 2.0.
*
- *In vitro* cleavage assay of *in vitro* transcribed RNAs with purified recombinant IRE1 cytosolic domains and denaturing urea-PAGE of IRE1-generated RNA fragments
  1. Assemble the *in vitro* cleavage reactions in non-stick 1.5 ml RNase-free tubes on ice.
  2. Assemble a master mix for your entire reaction time-series. Each time point has a final volume of 10 μl. Add the following components (in order; per reaction):
    - 2x RNA cleavage buffer
    - 15-100 ng of PAGE-purified *in vitro* transcribed RNA
    - 50 nM to 5 μM recombinant IRE1-KR43
  3. Place the tube containing the master mix in a thermomixer set at 30 °C* and incubate for predetermined amounts of time, ranging from 30 s to 90 min depending on the RNA you are studying. Do not agitate.
*Notes:*
    - Plan your time series. *You may use a dry bath or water bath set at 30 °C.
    - *Include a control for cleavage specificity by supplementing one duplicate reaction (the one corresponding to the longest time point) with 4μ8C to a final concentration of 10 μM. To inhibit IRE1, pre-incubate 10 μM IRE1-KR43 with 20 μM 4μ8C for 30 min on ice.*
  4. For each time point take out 7 μl from the master mix and stop the reactions by adding 1 μl of proteinase K solution (20 μg) and incubating for 10 min at 37 °C. Add 1 reaction volume of stop solution, mix gently and heat the samples to 80 °C; incubate for an additional 5 min, then cool the samples immediately on ice to prevent re-folding or base-pairing of the denatured RNA.
  5. Load 5 μl of reaction for each data point (~1/3 of the total volume per time point) on a Novex 6% TBE-Urea PAGE gels. Load diluted RNA ladder on the first well of the gel as indicated in step B6 “NOTE b”). Run the gel at 100 V (constant voltage) for 50-60 min (or longer if you are analyzing longer RNAs) using the XCell SureLock mini-cell electrophoresis system.
*Note: Before loading your samples, thoroughly rinse each well of the gel to remove urea buildup. Load your samples on the rinsed, clean wells.*
  6. Disassemble the XCell SureLock mini-cell, remove the gel and dismantle the cassette gel carefully, leaving the gel mounted on one of the plastic plates. Stain the gel with SYBR Gold (diluted as per manufacturer’s recommendations in 1x TBE) by submerging the gel in the buffer for 20 min at room temperature.
*Note: Avoid touching the gel with gloved fingers, as it will leave marks. Cover the gels to prevent exposure to light while staining.*
  7. Place the stained gel on the stage of a gel imaging system and capture digital images using various exposure times. Record the exposure times for each photograph. Use the longer exposure time that gives the best contrast without saturated pixels. Representative examples of typical results are shown in Figure 2.
    **Figure 2.**
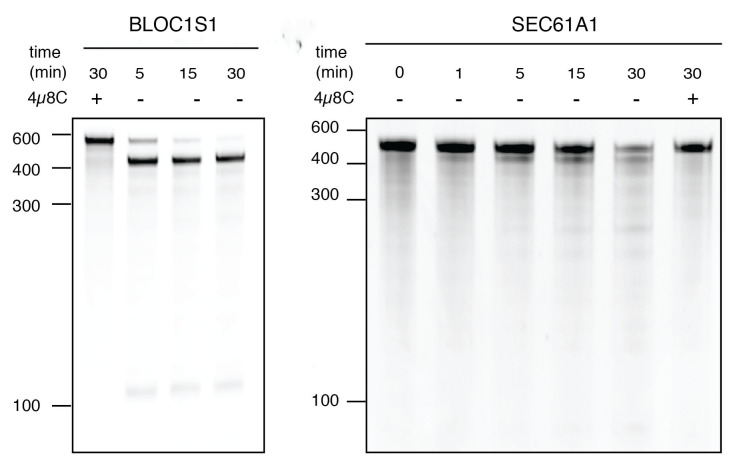
TBE-urea PAGE gels showing the IRE1-dependent cleavage of select IRE1 targets, BLOC1S1 (left) and SEC61A1 (right). 5 μM IRE1-KR43 was used to cleave 25 nM of RNA substrates for the indicated times. The IRE1 inhibitor 4µ8C was used as a control for specificity.

## Data analysis

The digital images of the gels can be densitometrically quantified and analyzed using ImageJ Software.

1. Adjust the image brightness and contrast and remove background prior to the analysis.
2. Draw boxes of equal size around the bands corresponding to the uncleaved RNA and calculate the area under the curve (pixel density).
3. Normalize your numbers to the uncleaved control and calculate the fraction of remaining uncleaved RNA. To determine observed rate constants, plot these values as a function of time and calculate the best curve fit using your choice of scientific graphing and analysis software.

## Recipes

*Notes:*

1. *For all recipes, use molecular biology grade reagents or pre-made solutions (available from multiple suppliers). Stock solutions should be prepared in RNase-free water.*
2. *To pH a 0.5 M or 1 M HEPES stock solution, use KOH pellets, add slowly while stirring the solution and monitor the pH continuously.*

*We have not observed significant differences when using Mg(OAc)_2_ as opposed to MgCl_2_.

1. SF21 cell lysis buffer
  20 mM HEPES pH 7.4
  600 mM KCl
2 mM MgCl_2_
  10% glycerol
  10 mM imidazole
  Supplement with freshly added cOmplete EDTA-free protease inhibitor cocktail
2. IRE1 dialysis buffer
  20 mM HEPES pH 7.4
  300 mM KCl
2 mM MgCl_2_
  5% glycerol
  1 mM TCEP-HCl
3. RNA gel extraction buffer
  300 mM NaOAc
  1 mM EDTA
  Supplemented with freshly added recombinant RNasin RNase inhibitor
4. RNA re-suspension buffer
  20 mM HEPES pH 7.4
  100 mM NaCl
1 mM MgCl_2_*
*Note: We have not observed significant differences when re-folding RNA in this buffer versus refolding in RNase-free water.*
5. 2x RNA cleavage buffer
  40 mM HEPES pH 7.4
  200 mM NaCl
2 mM MgCl_2_*
  10% glycerol
  2 mM TCEP-HCl
6. Stop solution
  10 M urea
  0.1% SDS
  1 mM EDTA
  0.01% xylene cyanol
  0.01% bromophenol blue
*Note: Bromophenol blue can generate background noise/signal artifacts during imaging when the RNA concentration is low.*
